# Machine learning-based identification of efficient and restrictive physiological subphenotypes in acute respiratory distress syndrome

**DOI:** 10.1186/s40635-025-00737-9

**Published:** 2025-03-01

**Authors:** Gabriela Meza-Fuentes, Iris Delgado, Mario Barbé, Ignacio Sánchez-Barraza, Mauricio A. Retamal, René López

**Affiliations:** 1 Instituto de Ciencias e Innovación en Medicina, Facultad de Medicina Clínica Alemana, Universidad del Desarrollo, Santiago, Chile; 2 Centro de Epidemiología y Políticas de Salud, Facultad de Medicina, Clínica Alemana, Universidad del Desarrollo, Santiago, Chile; 3 Programa de Comunicación Celular en Cáncer, Facultad de Medicina Clínica Alemana, Universidad del Desarrollo, Santiago, Chile; 4 Grupo Intensivo, ICIM, Facultad de Medicina, Clínica Alemana Universidad del Desarrollo, Santiago, Chile; 5 Departamento de Paciente Crítico, Clínica Alemana de Santiago, Santiago, Chile

**Keywords:** Acute respiratory distress syndrome, Subphenotypes, Artificial intelligence, Personalized medicine

## Abstract

**Introduction:**

Acute respiratory distress syndrome (ARDS) is a severe condition with high morbidity and mortality, characterized by significant clinical heterogeneity. This heterogeneity complicates treatment selection and patient inclusion in clinical trials. Therefore, the objective of this study is to identify physiological subphenotypes of ARDS using machine learning, and to determine ventilatory variables that can effectively discriminate between these subphenotypes in a bedside setting with high performance, highlighting potential utility for future clinical stratification approaches.

**Methodology:**

A retrospective cohort study was conducted using data from our ICU, covering admissions from 2017 to 2021. The study included 224 patients over 18 years of age diagnosed with ARDS according to the Berlin criteria and undergoing invasive mechanical ventilation (IMV). Data on physiological and ventilatory variables were collected during the first 24 h IMV. We applied machine learning techniques to categorize subphenotypes in ARDS patients. Initially, we employed the unsupervised Gaussian Mixture Classification Model approach to group patients into subphenotypes. Subsequently, we applied supervised models such as XGBoost to perform root cause analysis, evaluate the classification of patients into these subgroups, and measure their performance.

**Results:**

Our models identified two ARDS subphenotypes with significant clinical differences and significant outcomes. Subphenotype Efficient (*n* = 172) was characterized by lower mortality, lower clinical severity and presented a less restrictive pattern with better gas exchange compared to Subphenotype Restrictive (*n* = 52), which showed the opposite. The models demonstrated high performance with an area under the ROC curve of 0.94, sensitivity of 94.2% and specificity of 87.5%, in addition to an F1 score of 0.85. The most influential variables in the discrimination of subphenotypes were distension pressure, respiratory frequency and exhaled carbon dioxide volume.

**Conclusion:**

This study presents an approach to improve subphenotype categorization in ARDS. The generation of clustering and prediction models by machine learning involving clinical, ventilatory mechanics, and gas exchange variables allowed for more accurate stratification of patients. These findings have the potential to optimize individualized treatment selection and improve clinical outcomes in patients with ARDS.

**Graphical Abstract:**

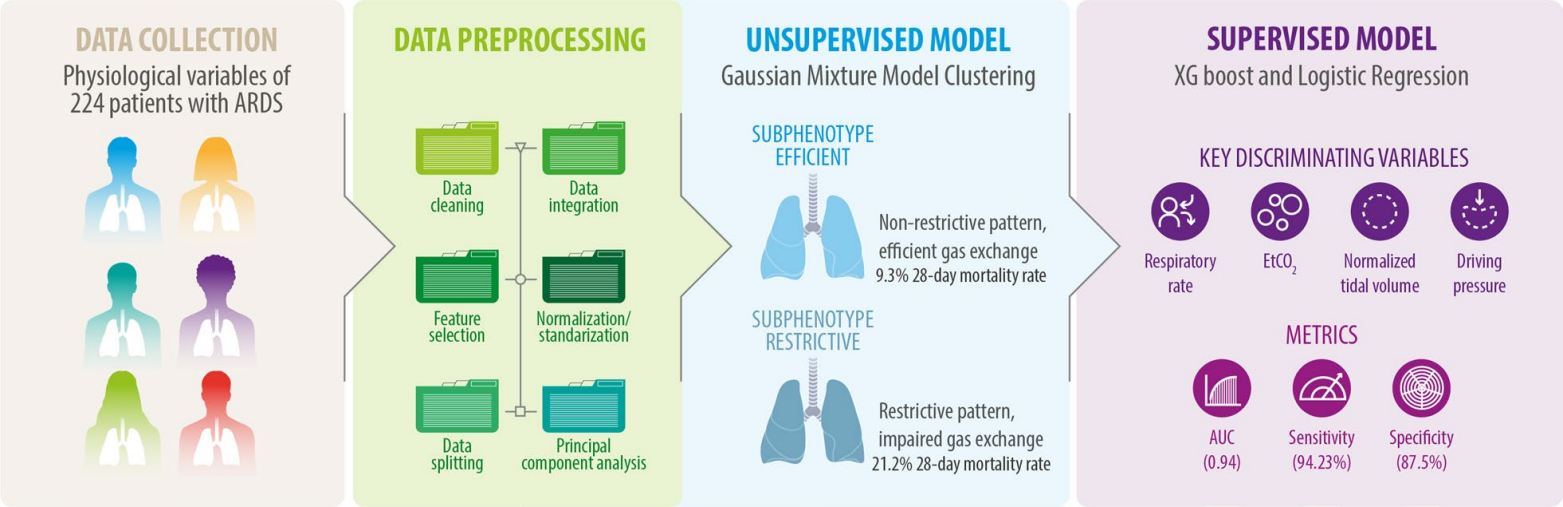

**Supplementary Information:**

The online version contains supplementary material available at 10.1186/s40635-025-00737-9.

## Introduction

Acute Respiratory Distress Syndrome (ARDS) is a life-threatening condition characterized by non-cardiogenic pulmonary edema resulting from increased alveolar-capillary membrane permeability [1]. ARDS represents a significant public health problem, accounting for 10.4% of ICU admissions globally and a 45% mortality rate among hospitalized patients [2].

One of the main challenges of ARDS is its clinical, radiological, pathological, and biological heterogeneity [3], complicating patient selection for clinical trials and personalized treatment adjustments [4], thus adding complexity to personalized treatment adjustments. While the Berlin definition has helped identify ARDS patients, many clinical trials, particularly those investigating pharmacological interventions, have failed [5].

The ARDS definition is currently under review [6], highlighting the need to refine patient categorization to optimize treatment strategies and clinical trial inclusion. Subphenotyping improves ARDS understanding by identifying patient groups with shared pathophysiology, which can guide treatment selection [7, 8]. However, there is no consensus on the best categorization model to adjust treatment or select patients for trials [4]. The classification proposed by the Berlin Consensus based on PaO2/FiO2 has a positive predictive validity for mortality with an area under the ROC curve of 0.57 [9], indicating poor discrimination of ARDS severity by this criterion alone.

Biomarkers have shown promise in ARDS diagnosis and stratification, but their clinical use remains limited due to small sample sizes and lack of validation [10, 11]. Meanwhile, protective mechanical ventilation strategies have been shown to improve survival by reducing mechanical stress on the lungs [12-14].

Incorporating physiological variables into ARDS patient evaluation is crucial for improving subphenotyping, as it allows a more accurate understanding of the disease's complexity [15]. Machine learning techniques have proven useful in classifying ARDS subphenotypes using readily obtainable bedside physiological data, enabling personalized treatment and better clinical outcomes [16, 17].

Therefore, the aim of this study was to develop a machine learning-based mechanism for subphenotyping ARDS patients using physiological variables to facilitate personalized medicine.

## Methodology

### Study design

A retrospective cohort study was conducted using data prospectively collected from the Intensive Care Unit (ICU) of Clínica Alemana de Santiago, with approval from the Institutional Ethics Committee (IRB #2012-53). The informed consent was waived in agreement with the observational nature of this study. The procedures were followed in accordance with the ethical standards of the responsible committee on human experimentation (institutional or regional) and with the Helsinki Declaration of 1975. Secondary databases of institutional hospital records collected between 2017 and 2021 were validated, integrated, and standardized. Values of variables during the first 24 h of ICU admission were collected.

### Patient selection

Patients were selected according to the following inclusion criteria: individuals over 18 years of age, hospitalized in our ICU, undergoing invasive mechanical ventilation, meeting the Berlin criteria for the diagnosis of ARDS [9]. Additionally, all patients included in the study were managed using volume-controlled ventilation (VCV) as the primary mode during the first 24 h post-intubation. This standardized approach ensured consistency in the measurement of key ventilatory mechanics [10][18]. Exclusion criteria included lung transplant recipients, individuals with chronic respiratory diseases, patients requiring treatment with extracorporeal membrane oxygenation (ECMO), and those in spontaneous ventilation mode.

### Data preprocessing

After the consolidation and standardization of secondary databases, appropriate variables for machine learning models were selected, eliminating redundant and collinear variables. Pearson correlation coefficients were calculated to identify highly collinear pairs (correlation coefficient > 0.8), and one variable from each pair was removed based on its clinical interpretability.

To address redundancy, each variable was evaluated using *R*^2^ values to quantify its predictability by other variables. Variables with high redundancy were excluded if they contributed minimal unique information. Following this selection process, a dimensionality reduction technique, specifically principal component analysis (PCA), was applied to address multicollinearity, reduce dimensionality, and enhance cluster separability. The components derived from PCA were subsequently used for clustering with the Gaussian Mixture Model (GMM).

### Machine learning approaches utilized for patient subgroup identification and classification

First, the data were clustered, starting with the evaluation to determine the optimal number of clusters. The elbow method, Akaike Information Criterion (AIC), and silhouette scoring were employed as complementary metrics to assess intra-cluster cohesion and inter-cluster separation. These metrics confirmed that two clusters best represented the data structure (*k* = 2) (Supplementary Figure S1).

Then, a Gaussian Mixture Model (GMM) was applied for clustering the data, using the PCA-transformed components as input. The GMM-EM algorithm was initialized with centroids generated by K-Means clustering to ensure stability in the iterative refinement of clusters.

Using the labels obtained from the GMM, supervised models were developed. For this purpose, the dataset was divided into training and test sets in a 70:30 ratio. Recursive Feature Elimination with Cross-Validation (RFECV) was performed after clustering to select the most relevant features for classification, leveraging an XGBoost classifier. XGBoost was selected based on the highest performance. XGBoost was used to evaluate the model metrics and to select the variables that most influence the classification. The supervised models were developed using libraries from scikit-learn [11] [19] and AutoML PyCaret [20] to perform root cause analysis and evaluate the classification of patients into these subgroups.

Finally, a parsimonious supervised classification model was developed using linear regression, generating a score based on the odds ratios of the identified variables.

The machine learning workflow followed a structured sequence of steps, including preprocessing, clustering, and supervised modeling**.** A detailed workflow diagram is provided in Supplementary Figure S2, summarizing each step from raw data preprocessing to the final parsimonious model development.

### Statistical analysis

Descriptive statistics were used to summarize variables, with medians and interquartile ranges for continuous variables, and frequencies and percentages for categorical variables.

To compare baseline characteristics between subphenotypes, Chi-square tests were utilized for categorical variables, while Mann–Whitney *U* tests were applied for continuous variables due to the non-normal distribution of the data.

A significance level (alpha) of 0.005 and a power of 80% were established for the analysis.

## Results

**Fig. 1:**
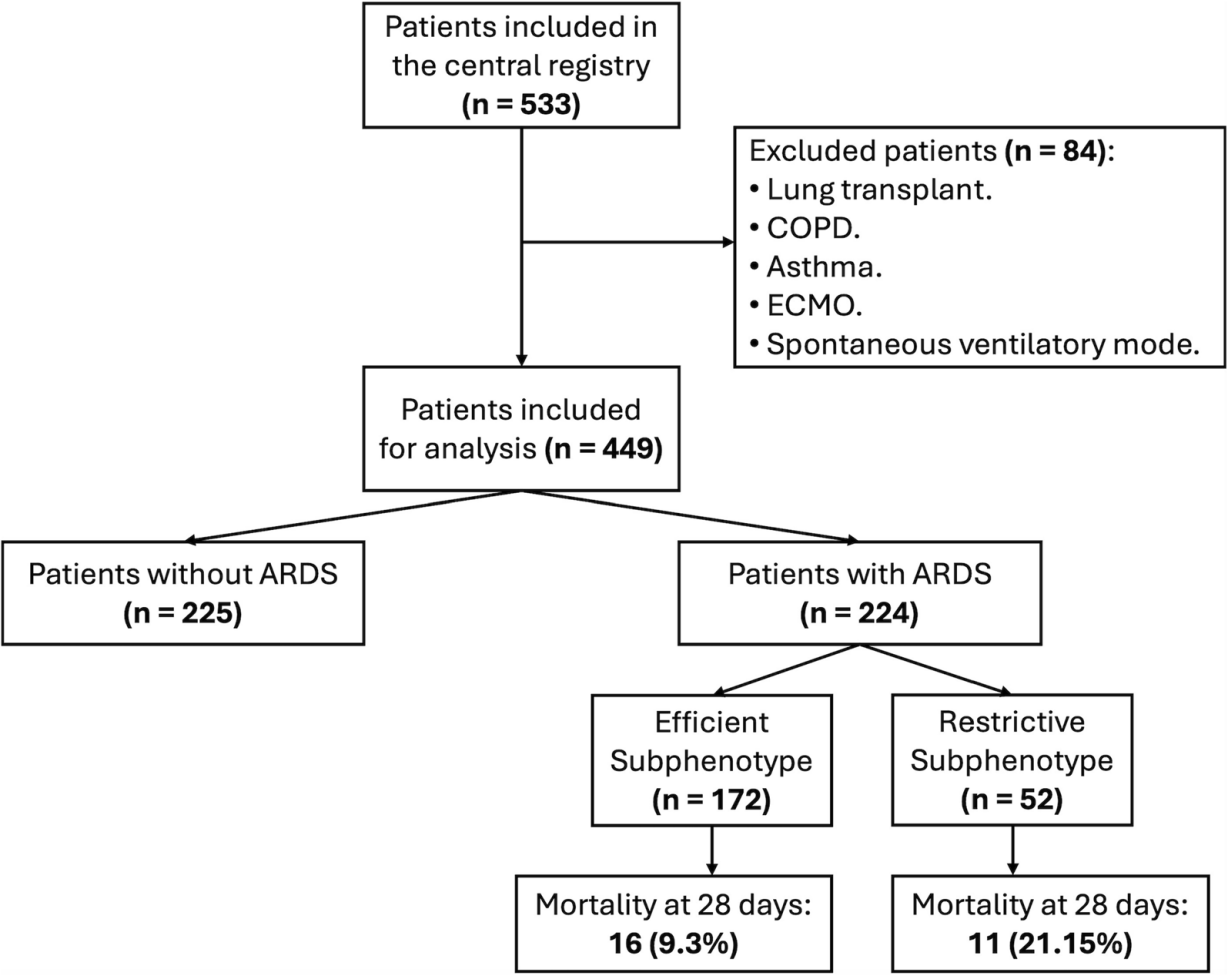
Study flowchart

A total of 533 patients were identified, of whom 224 had ARDS according to the Berlin definition. These patients were analyzed for subphenotyping (see flowchart in Fig. 1). Overall, the cohort consisted predominantly of male patients with moderate severity based on APACHE II and SOFA scores, and moderate ARDS according to oxygenation and Lung Injury Score. The primary reason for admission was medical in nature, and approximately one-third of the patients had sepsis or shock (see Table 1).

**Table 1:** Baseline characteristics

Baseline characteristics	
Age (years)	57 (46–66)
Male (gender)	158 (70.54%)
Size (m)	1.70 (1.64–1.77)
IMC (kg/cm^2^)	27.69 (25.14–31.44)
PBW (kg)	71.02 (61.92–75.57)
*Admission type*	
Medical	205 (91.52%)
Surgical	13 (5.8%)
Neurocritical	5 (2.23%)
Trauma	1 (0.45%)
*Source of admission*	
Urgency	144 (64.29%)
Other	80 (35.71%)
Sepsis at the ICU admission	36 (16.07%)
Shock at the ICU admission	61 (27.23%)
ICU stay (days)	9 (6–15)
*Severity of illness*	
SOFA	6 (4–7)
LIS	2.75 (2.25–3)
APACHE II at the ICU admission	9 (5–15)
*Laboratory data at the beginning of ventilation*	
PaO2/FiO2 (mmHg)	194.44 (140.91–252)
PaCO2 (mmHg)	38.8 (35.1–43.2)
Lactic acid (mg/dL)	15.6 (12.4–20.6)

*BMI* body mass index, *PBW* predicted body weight, *SOFA* Sequential Organ Failure Assessment, *LIS* Lung Injury Score, *APACHE II* Acute Physiology and Chronic Health Evaluation II. Results are presented as median and interquartile range (IQR)

### Principal component analysis (PCA) and variable selection

Principal component analysis revealed that 90% of the data variability could be explained by 6 dimensions. Following this analysis, a set of variables was selected for further investigation. These variables included total positive end-expiratory pressure (PEEP), peak pressure, plateau pressure, respiratory rate, minute volume, end-tidal carbon dioxide (EtCO_2_, carbon dioxide production (VCO_2_), partial pressure of carbon dioxide (PaCO_2_), partial pressure of oxygen (PaO_2_), partial pressure to inspired fraction of oxygen ratio (PaO_2_/FiO_2_), driving pressure, normalized tidal volume, normalized mechanical power, static compliance of respiratory system and respiratory dead space (VD/VT).

### Gaussian mixture model clustering approach

**Fig. 2:**
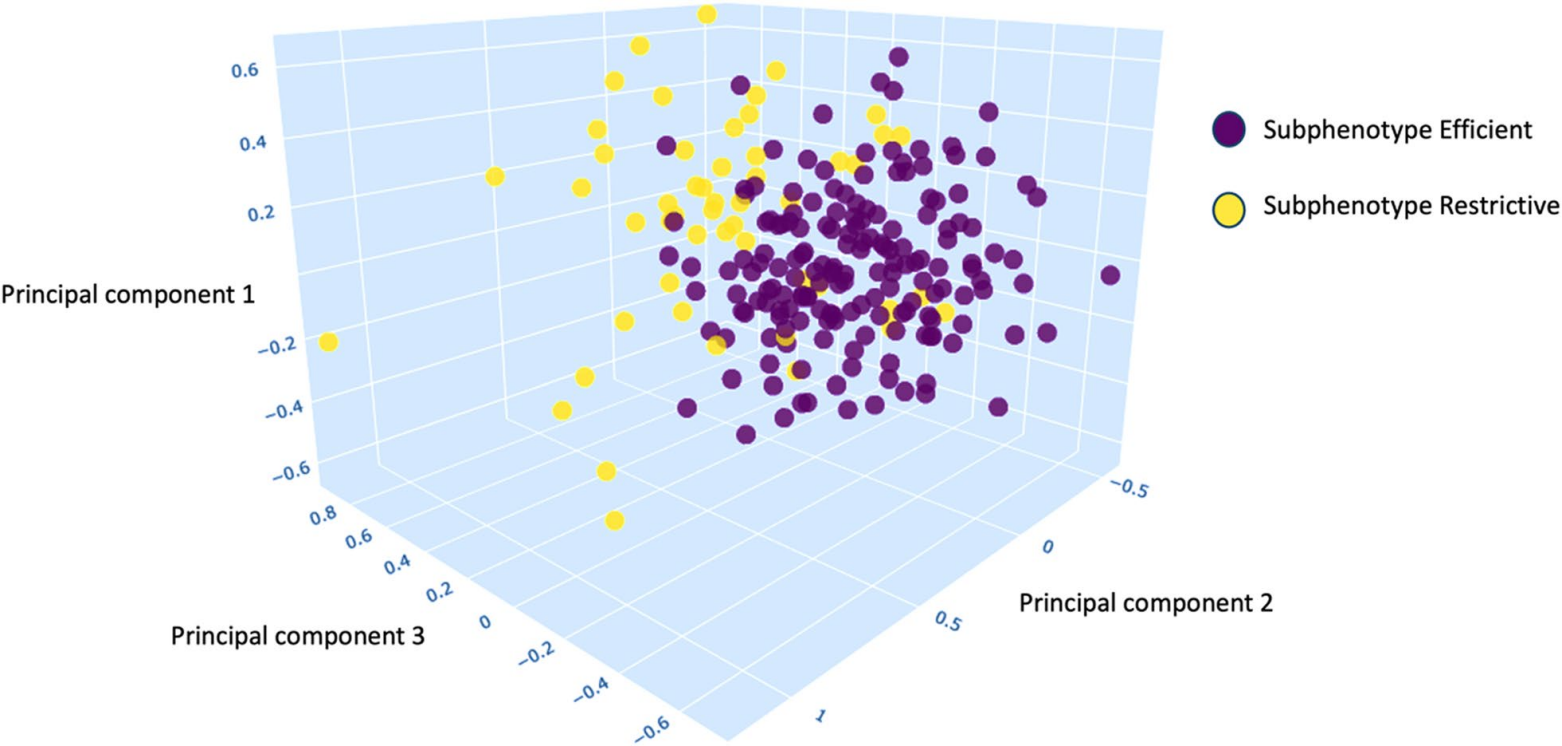
Clustering approach using Gaussian Mixture Model (GMM). A three-dimensional representation of the three main components obtained through principal component analysis (PCA). Each point represents a patient, and the colors indicate the assigned subphenotype

Using Gaussian Mixture Model (GMM), it was determined that only two subphenotypes provided the best fit to the observed data (Fig. 2). These subphenotypes were named termed “efficient” and “restrictive” to reflect their primary characteristics based on ventilatory efficiency and mechanical properties.

The “Efficient” subphenotype consisted of 172 patients, while the “Restrictive” subphenotype comprised 52 patients. There were no significant differences observed in age, gender distribution, height, BMI, or predicted body weight between the two subphenotypes (Table 2). However, significant differences were noted in several outcomes and disease severity parameters. Subphenotype Efficient exhibited lower 28-day mortality rates (9.3% vs. 21.15%, *p* = 0.021), shorter ICU stays (9 vs. 10 days, *p* = 0.387), and lower severity scores including SOFA (6 vs. 7, *p* = 0.004), and APACHE II (9 vs. 12, *p* = 0.01) indicating a less severe clinical presentation compared to Subphenotype Restrictive. Additionally, Subphenotype Efficient demonstrated a less restrictive ventilatory behavior compared to Subphenotype Restrictive, characterized by higher tidal volume normalized to predicted body weight (4.37 vs. 3.84 ml/kg/PBW, *p* < 0.001), lower plateau pressure (21 vs. 24 cmH2O, *p* < 0.001), lower static compliance (40 vs. 31.08 cmH2O/L, *p* < 0.001), and lower driving pressure (10 vs. 11 cmH2O, *p* < 0.001) compared to Subphenotype Restrictive. Moreover, Subphenotype Efficient exhibited greater ventilatory efficiency, as evidenced by lower dead space to tidal volume ratio (0.5 vs. 0.62, *p* < 0.001), lower end-tidal carbon dioxide levels (30.5 vs. 36 mmHg, *p* < 0.001), lower respiratory rate (24 vs. 30 rpm, *p* < 0.001), lower partial pressure of carbon dioxide (38.1 vs. 42.5 mmHg, *p* < 0.001), and lower minute ventilation (8.8 vs. 10.55 l/min, *p* < 0.001) compared to Subphenotype Restrictive. Furthermore, Subphenotype Efficient had lower mechanical power (0.19 vs. 0.24 × 10–3 J/min/kg, *p* < 0.001) compared to Subphenotype Restrictive.

**Table 2:** Baseline characteristics of the included patients

	Subphenotype efficient (*n* = 172)	Subphenotype restrictive (*n* = 52)	*p*-value
*Baseline characteristics*			
Age (years)	58 (46–66)	56 (42–67)	0.554
Male (gender)	123 (71.51%)	35 (67.31%)	0.56
Size (m)	1.7 (1.64–1.78)	1.7 (1.62–1.75)	0.443
BMI (kg/cm^2^)	27.68 (25.22–31.14)	27.76 (24.8–32.83)	0.711
PBW (kg)	71.02 (65.21–75.32)	71.02 (65.48–75.5)	0.63
*Disease severity*			
SOFA	6 (3–7)	7 (5–10)	0.004*
LIS	2.75 (2.25–3)	2.75 (2.5–3.25)	0.04*
APACHE II	9 (5–13)	12 (5–19)	0.01*
28-Day Mortality	16 (9.3%)	11 (21.15%)	0.021*
*Treatment*			
Neuromuscular block	116 (78.38%)	42 (93.33%)	0.023*
Prone	73 (42.44%)	23 (44.23%)	0.819
*First day of ventilation*			
Mechanical power (J/min)	17.21 (14.11–19.84)	21.61 (17.58–29.09)	< 0.001*
Tidal volume (ml/kg/PBW)	4.37 (4.02–4.82)	3,84 (2.96–4.59)	< 0.001*
Plateau pressure (cmH2O)	21 (18–23)	24 (20–26)	< 0.001*
Driving pressure (cmH2O)	10 (8–11)	11 (10–12)	< 0.001*
Total respiratory rate (rpm)	24 (20–26)	30 (26–36)	< 0.001*
Minute ventilation (l/min)	8.8 (7.6–9.8)	10.55 (8.85–11.75)	< 0.001*
VD/VT	0.5 (0.43–0.57)	0.62 (0.54–0.73)	< 0.001*
norMP (× 10–3 J/min/kg)	0.19 (0.16–0.23)	0.24 (0.2–0.33)	< 0.001*
Static compliance (cmH2O/L)	40 (34.55–47.5)	31.08 (25.23–38.54)	< 0.001*
EtCO2 (mmHg)	30.5 (33–35.5)	36 (34–41)	< 0.001*
PaCO2 (mmHg)	38.1 (34.1–41.5)	42.5 (38.6–49)	< 0.001*
PEEP (cmH2O)	12 (10–14)	13 (10–15)	0.446
PaO2/FiO2 (%)	197.67 (145–258)	177.05 (113.4–248)	0.092

*BMI* body mass index, *PBW* predicted body weight, *SOFA* Sequential Organ Failure Assessment, *LIS* Lung Injury Score, *APACHE II* Acute Physiology and Chronic Health Evaluation II, VD/VT respiratory dead space, norMP normalized Mechanical Power, EtCO2 end-tidal carbon dioxide, PEEP positive end-expiratory pressure. Results are presented as median and interquartile range (IQR)

It is worth mentioning that positive end-expiratory pressure (PEEP) (12 vs. 13 cmH2O, *p* = 0.446) and the ratio of partial pressure of oxygen to fraction of inspired oxygen (PaO2/FiO2) (197.67 vs. 177.05, *p* = 0.092) were the only ventilatory variables that did not show significant differences between the two subphenotypes.

### Supervised model: XGBoost

**Fig. 3:**
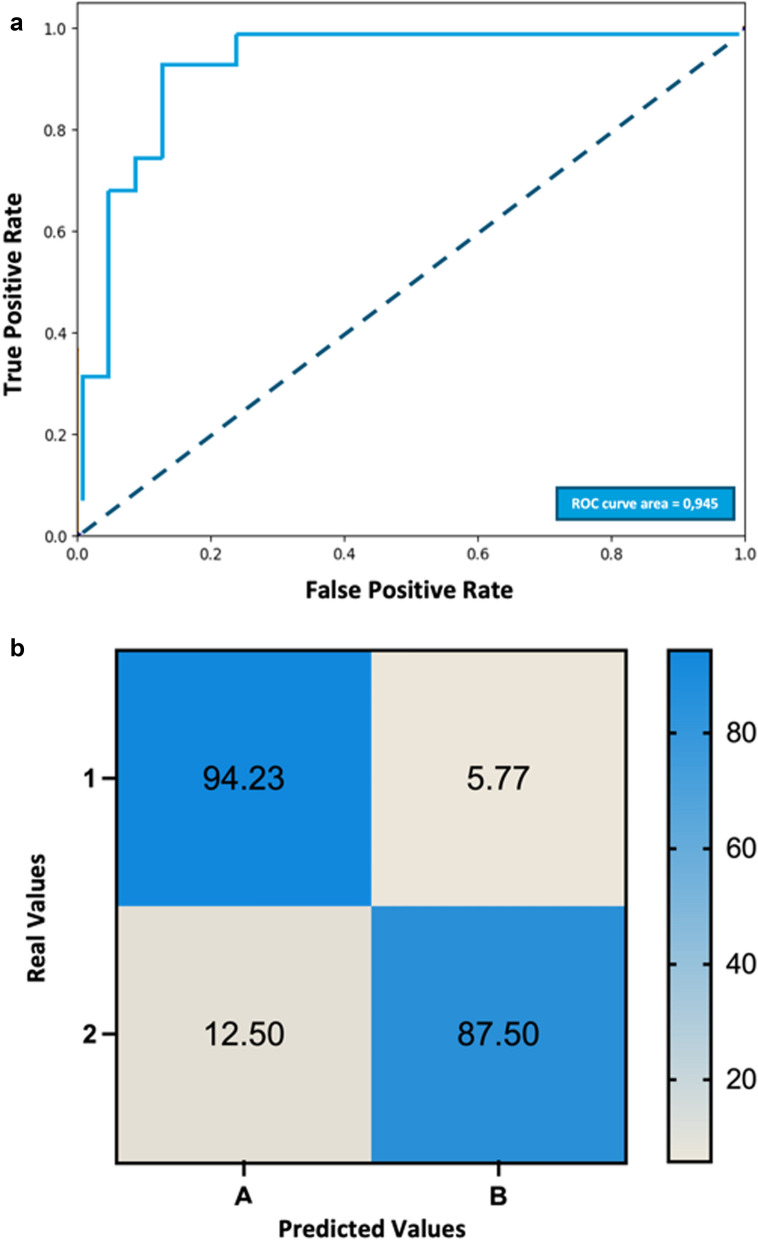
Performance of the XGBoost supervised model. **a** ROC curve demonstrating the performance of the XGBoost supervised model for classifying subphenotypes in ARDS patients. The area under the curve (AUC) is 0.94. **b** Sensitivity of 94.23% and specificity of 87.5%

**Fig. 4:**
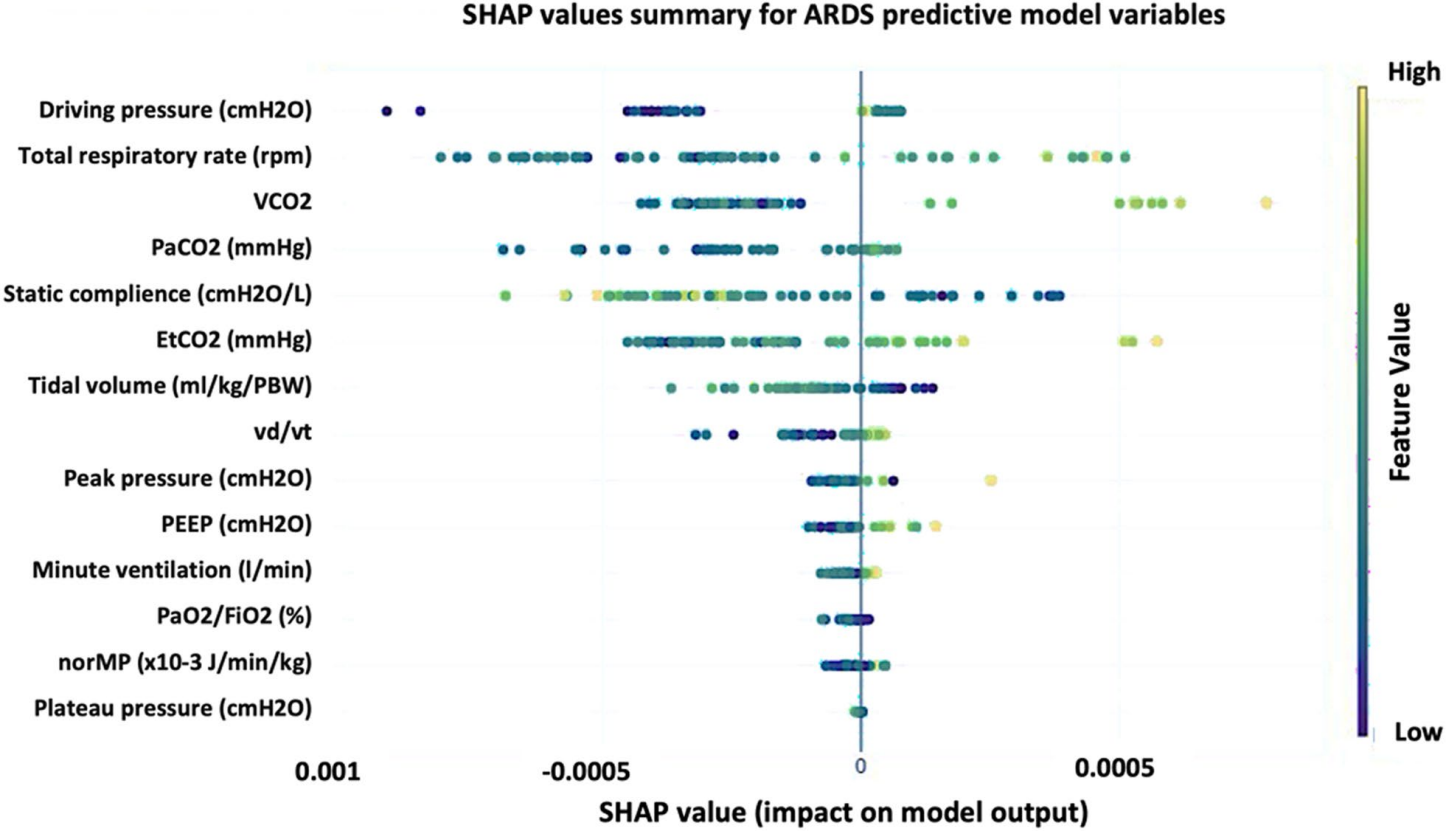
SHAP values summary for ARDS predictive model variables. This figure illustrates the importance of each variable in the prediction model, showing the impact of individual variables on the model output

In order to refine our model, we used a supervised models to further analyze the data. The subphenotypes identified in the unsupervised Gaussian Mixture Model were used as labels for training the XGBoost model. The XGBoost supervised model exhibited excellent performance, achieving an area under the ROC curve of 0.94, sensitivity of 94.23%, and specificity of 87.5% (Fig. 3). Additionally, SHAP (SHapley Additive exPlanations) values were analyzed to determine the most influential variables in the model. The SHAP summary plot (Fig. 4) shows the importance of each variable in the prediction model, highlighting their impact on the model output.

### Supervised model: logistic regression

A simplified logistic regression model was developed to identify the most influential variables for subphenotype discrimination and to create a parsimonious model. The model's performance was evaluated using several metrics. The Model Likelihood Ratio Test yielded a *χ*2 of 143.44 (*d.f.* = 8, Pr(> *χ*2) < 0.0001), with an *R*2 of 0.715. Discrimination indexes included a C-statistic of 0.948, indicating excellent model performance. Rank discrimination indexes showed a Dxy of 0.896, *γ* of 0.896, and τa of 0.321. The Brier score for the model was 0.064, indicating good calibration. The maximum absolute partial derivative of the log-likelihood with respect to *β* was 0.0003, demonstrating the model's stability.

**Fig. 5:**
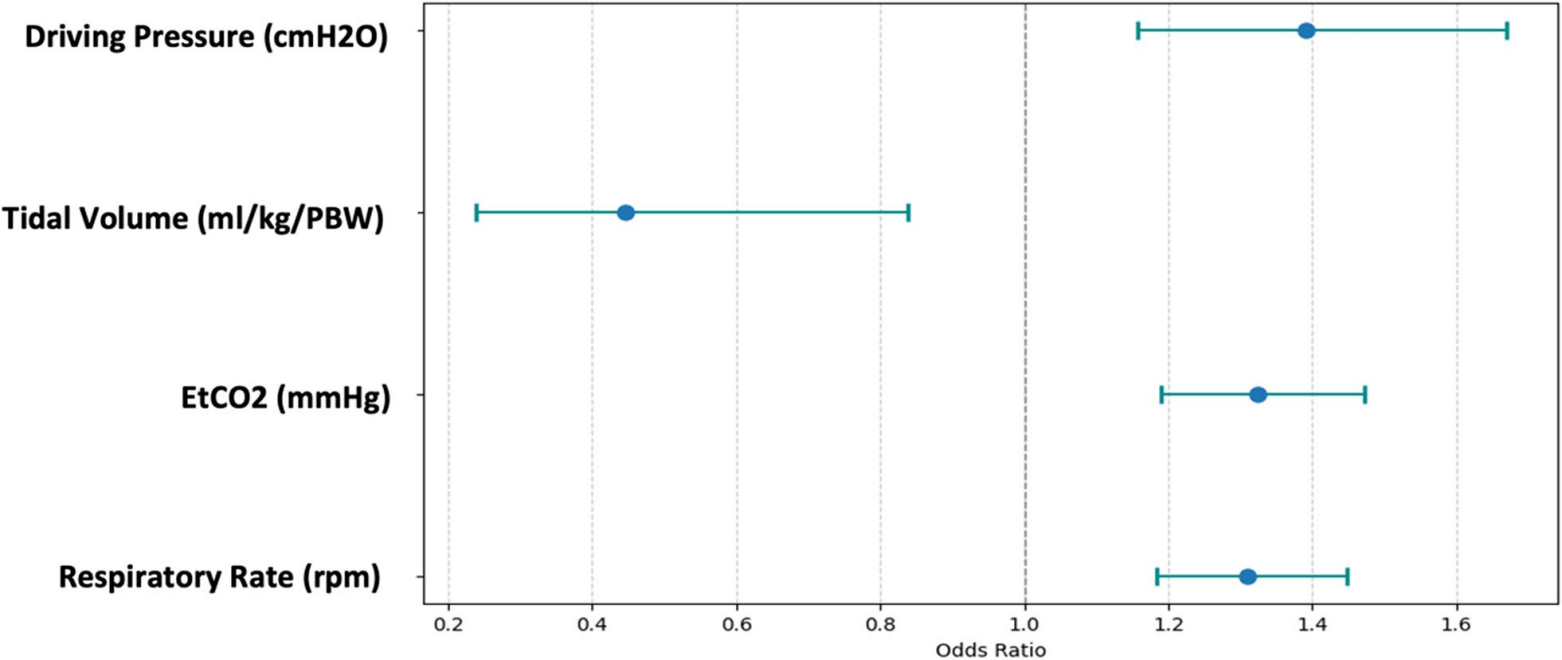
Most influential variables for subphenotype discrimination based on odds ratios. Variable importance plot based on the logistic regression model, showing the odds ratios of each variable. EtCO_2_ = end-tidal CO_2_

The analysis revealed that respiratory rate (*β* = 0.27, OR = 1.31, 95% CI [1.18, 1.45], *p* < 0.001), EtCO2 (*β* = 0.28, OR = 1.32, 95% CI [1.19, 1.47], *p* < 0.001), tidal volume (*β* = − 0.81, OR = 0.45, 95% CI [0.24, 0.84], *p* = 0.01), and driving pressure (*β* = 0.33, OR = 1.39, 95% CI [1.16, 1.67], *p* < 0.001) were significant predictors of ARDS subphenotypes (Table 3). To complement, Fig. 5 illustrates the odds ratios for these predictor variables, providing a visual representation of their impact and significance in the model.

**Table 3:** Multivariate logistic regression analysis for predicting physiological subphenotypes in ARDS patients

Predictor	Multivariate model				
	*β*	*p*-value	OR	95% CI lower	95% CI upper
Respiratory rate (rpm)	0.269	< 0.001	1.309	1.184	1.448
EtCO2 (mmHg)	0.28	< 0.001	1.324	1.189	1.473
Tidal volume (ml/kg/PBW)	− 0.806	0.012	0.446	0.238	0.838
Driving pressure (cmH2O)	0.33	< 0.001	1.391	1.158	1.67

*β* Beta coefficient, *OR* odds ratio, *CI* confidence interval, *PBW* predicted body weight. Statistical significance set at *p* < 0.05

**Fig. 6:**
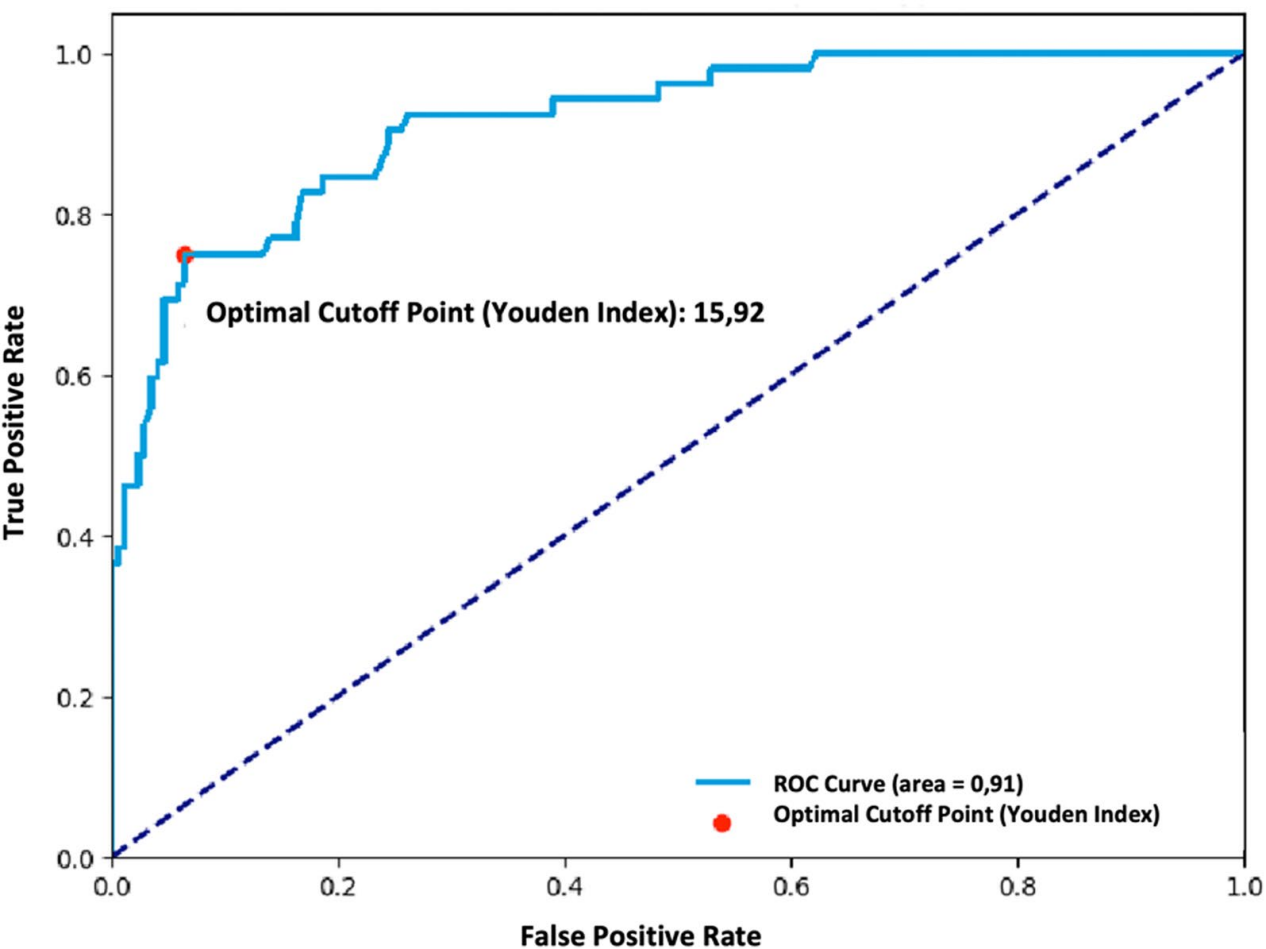
Classification score generated from weighted variables. Chart showing the discriminatory power of the classification score based on weighted variables. The score has a sensitivity of 0.75, a specificity of 0.94, and an area under the ROC curve of 0.91

To enhance the practical utility of our model, we created a classification score based on the weighted odds ratios of these variables. This score demonstrated high discriminatory power, with a cutoff of 16 according to the Youden index of the ROC curve. The classification score yielded a sensitivity of 0.75, specificity of 0.94, and an area under the ROC curve of 0.91 (Fig. 6).

## Discussion

### Identification of physiological subphenotypes in ARDS

Our study identified distinct physiological subphenotypes within the ARDS population, highlighting the heterogeneity of the disease. Patients with more restrictive ventilatory mechanics and higher mechanical power exhibited greater lung injury severity and a more severe ARDS presentation. This aligns with prior research showing the association between elevated mechanical power and increased lung injury severity [21]. In addition, we found significantly higher static compliance and driving pressure in more severe patients (Subphenotype Restrictive), supporting evidence that higher driving pressure is linked to reduced survival [22]. Lower tidal volumes are associated with lower mortality [13], and patients in Subphenotype Efficient tolerated higher tidal volumes, suggesting a less severe condition.

Furthermore, patients exhibiting poorer ventilatory efficiency are known to face increased mortality risk in the early stages of ARDS [23]. Specifically, increased dead-space fraction, such as the dead space-to-tidal volume ratio (VD/VT) [24] or ventilatory ratio [25], and end-tidal alveolar dead space fraction [26] have been associated with elevated mortality rates. In our study, variables such as dead space (VD/VT), VCO_2_, and PaCO_2_ were significantly higher in Subphenotype Restrictive, with EtCO_2_ emerging as the most relevant discriminator among all ventilatory efficiency variables. These findings are consistent with clinical evidence and existing literature, providing another perspective for identifying physiological subphenotypes in an easy, reproducible, and bedside manner, while elucidating the most relevant variables for subphenotype discrimination.

The names of the subphenotypes reflect their mechanical and efficiency characteristics, though they remain arbitrary and do not imply absolute classification.

### Clinical implications

The identification of subphenotypes in ARDS has important clinical implications. Subphenotype Efficient was associated with lower mortality and less severe clinical features, whereas Subphenotype Restrictive exhibited characteristics of more severe lung injury. These differences highlight the importance of future studies evaluating whether treatment strategies should differ between these subphenotypes. Variables such as driving pressure, respiratory rate, and exhaled CO₂ were key in discriminating between subphenotypes, offering potential avenues for targeted research. Previous studies have shown differential treatment responses among subphenotypes, with hyperinflammatory subphenotypes responding better to PEEP and recruitable phenotypes benefiting from higher PEEP levels [27]. The mechanical behavior observed in Subphenotype Restrictive suggests the need for further research into how interventions, such as higher PEEP or protective mechanical ventilation, may affect outcomes.

### Limitations and future directions

Despite the study's strengths, several limitations warrant consideration. The retrospective nature of the analysis and reliance on data from a single center may limit the generalizability of findings to broader ARDS populations. Additionally, while machine learning techniques enable comprehensive data analysis, further validation in prospective multicenter studies is necessary to confirm the reproducibility and external validity of identified subphenotypes. Future research endeavors should focus on integrating additional clinical and biological variables, refining subphenotype classification models, and evaluating the heterogeneity of treatment effects to assess whether specific interventions could be tailored to subphenotype characteristics.

## Conclusion

In conclusion, this study demonstrates the feasibility and effectiveness of applying machine learning techniques to identify subphenotypes in ARDS patients using variables from respiratory physiology. The delineation of these subphenotypes reveals significant clinical and outcome differences, emphasizing the importance of precise stratification to enhance personalized treatment. By elucidating distinct physiological subgroups, the study provides valuable insights into the pathophysiological mechanisms underlying ARDS and underscores the potential for tailored therapeutic approaches to optimize treatment selection and improve clinical outcomes. Moving forward, the integration of advanced subphenotyping techniques into clinical practice holds promise for revolutionizing ARDS management, facilitating more precise patient stratification, and ultimately improving patient outcomes.

## Supplementary Information

Supplementary Material 1. Figure S1. Evaluation of the optimal number of clusters using Silhouette scores, Elbow method, and Akaike Information Criterion. The Silhouette method, Elbow method, and Akaike/Bayesian Information Criterionwere used to assess the optimal number of clusters. The best cluster numberis indicated by the red dashed line. Figure S2. Workflow for machine learning approaches. A detailed schematic illustrating the workflow from data preprocessing to the development of the final parsimonious linear model. Each step highlights key decisions and processes involved.

## Data Availability

The datasets generated and/or analyzed during the current study are not publicly available due to ethical restrictions concerning patient privacy and confidentiality, but are available from the corresponding author on reasonable request.
