# Supplementary material for: Machine learning-based identification of efficient and restrictive physiological subphenotypes in acute respiratory distress syndrome
Source: Intensive Care Med Exp. 2025 Mar 1;13:29. doi: 10.1186/s40635-025-00737-9 (PMC11872963; doi:10.1186/s40635-025-00737-9)
Supplement: Supplementary file 1 — Supplementary Material 1. Figure S1. Evaluation of the optimal number of clusters using Silhouette scores, Elbow method, and Akaike Information Criterion. The Silhouette method, Elbow method, and Akaike/Bayesian Information Criterionwere used to assess the optimal number of clusters. The best cluster numberis indicated by the red dashed line. Figure S2. Workflow for machine learning approaches. A detailed schematic illustrating the workflow from data preprocessing to the development of the final parsimonious linear model. Each step highlights key decisions and processes involved. [file 40635_2025_737_MOESM1_ESM.docx]

# Supplementary Material

# Supplementary Figure S1. Evaluation of the optimal number of clusters using Silhouette scores, Elbow method, and Akaike Information Criterion (AIC).

#
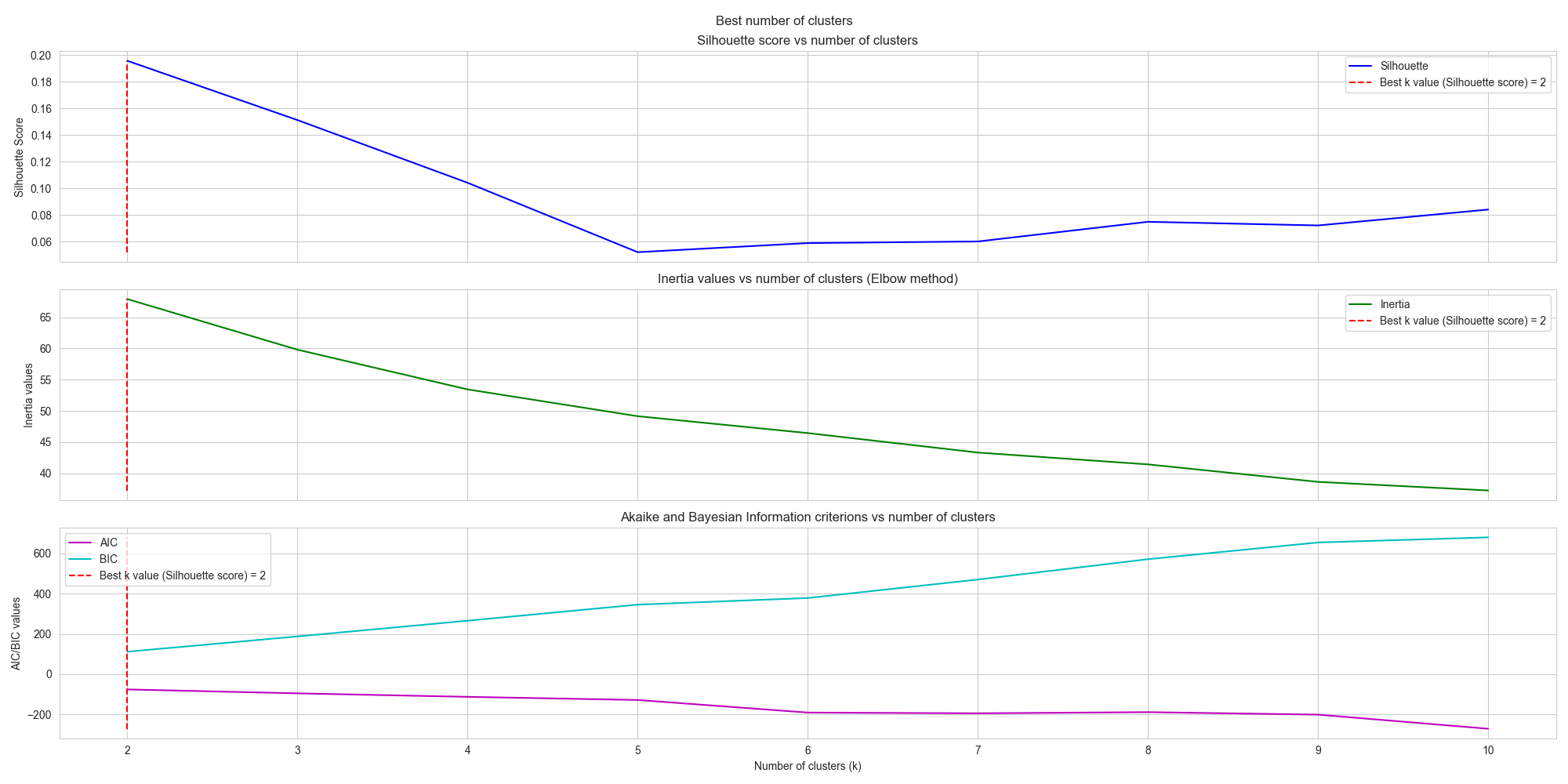


# The Silhouette method (top), Elbow method (middle), and Akaike/Bayesian Information Criterion (bottom) were used to assess the optimal number of clusters. The best cluster number (*k*=2) is indicated by the red dashed line.

# Supplementary Figure S2. Workflow for machine learning approaches.

#
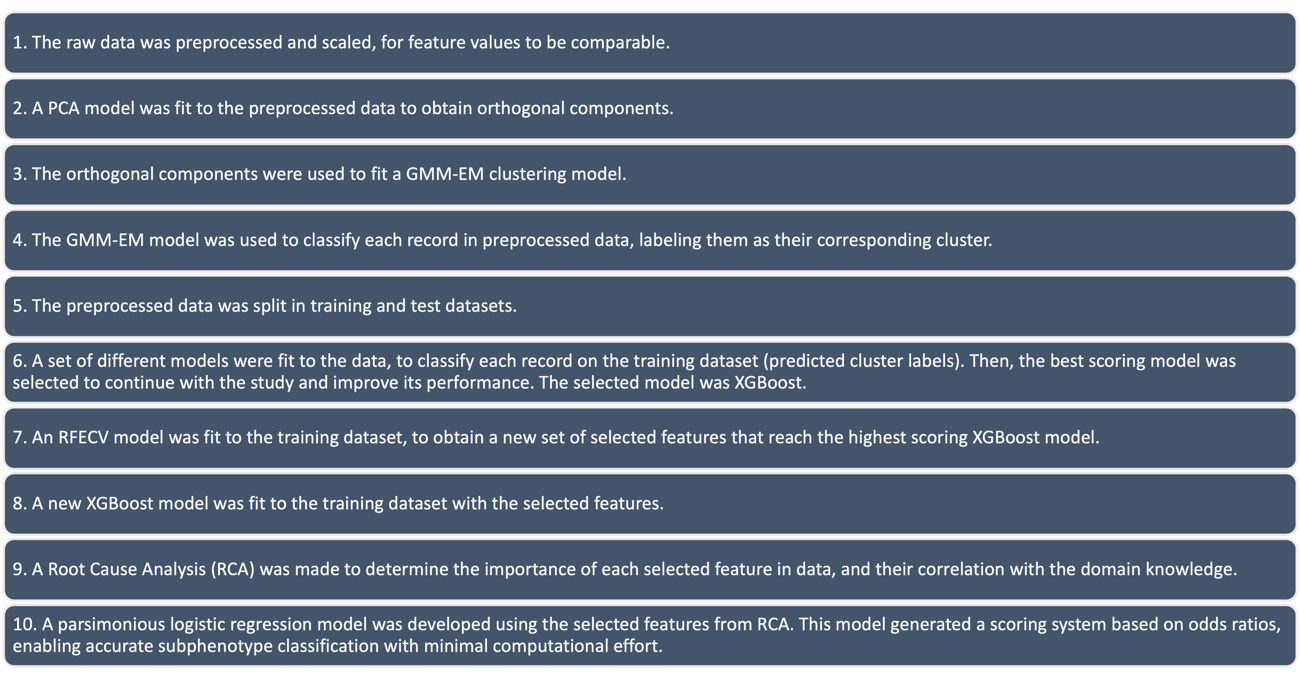


# A detailed schematic illustrating the workflow from data preprocessing to the development of the final parsimonious linear model. Each step highlights key decisions and processes involved.
